# Fraud in Animal Origin Food Products: Advances in Emerging Spectroscopic Detection Methods over the Past Five Years

**DOI:** 10.3390/foods9081069

**Published:** 2020-08-06

**Authors:** Abdo Hassoun, Ingrid Måge, Walter F. Schmidt, Havva Tümay Temiz, Li Li, Hae-Yeong Kim, Heidi Nilsen, Alessandra Biancolillo, Abderrahmane Aït-Kaddour, Marek Sikorski, Ewa Sikorska, Silvia Grassi, Daniel Cozzolino

**Affiliations:** 1 Nofima AS, Norwegian Institute of Food, Fisheries, and Aquaculture Research, Muninbakken 9-13, 9291 Tromsø, Norway; ingrid.mage@Nofima.no (I.M.); heidi.nilsen@nofima.no (H.N.); 2 United States Department of Agriculture, Agricultural Research Service, 10300 Baltimore Avenue, Beltsville, MD 20705-2325, USA; walter.schmidt@usda.gov; 3 Department of Food Engineering, Bingol University, 12000 Bingol, Turkey; tumaytemiz89@gmail.com; 4 Key Laboratory of Mariculture, Ministry of Education, Ocean University of China, Qingdao 266003, China; l_li@ouc.edu.cn; 5 Department of Food Science and Biotechnology, Kyung Hee University, Yongin 17104, Korea; hykim@khu.ac.kr; 6 Department of Physical and Chemical Sciences, University of L’Aquila, 67100 Via Vetoio, Coppito, L’Aquila, Italy; alessandra.biancolillo@univaq.it; 7 Université Clermont-Auvergne, INRAE, VetAgro Sup, UMR F, 63370 Lempdes, France; abderrahmane.aitkaddour@vetagro-sup.fr; 8 Faculty of Chemistry, Adam Mickiewicz University in Poznan, Uniwersytetu Poznanskiego 8, 61-614 Poznan, Poland; sikorski@amu.edu.pl; 9 Institute of Quality Science, Poznań University of Economics and Business, al. Niepodległości 10, 61-875 Poznań, Poland; ewa.sikorska@ue.poznan.pl; 10 Department of Food, Environmental and Nutritional Sciences (DeFENS), Università degli Studi di Milano, via Celoria, 2, 20133 Milano, Italy; silvia.grassi@unimi.it; 11 Centre for Nutrition and Food Sciences, Queensland Alliance for Agriculture and Food Innovation, The University of Queensland, 39 Kessels Rd, Coopers Plains, QLD 4108, Australia; d.cozzolino@uq.edu.au

**Keywords:** authentication, authenticity, chemometric, fish, origin, honey, meat, milk, spectroscopy, species

## Abstract

Animal origin food products, including fish and seafood, meat and poultry, milk and dairy foods, and other related products play significant roles in human nutrition. However, fraud in this food sector frequently occurs, leading to negative economic impacts on consumers and potential risks to public health and the environment. Therefore, the development of analytical techniques that can rapidly detect fraud and verify the authenticity of such products is of paramount importance. Traditionally, a wide variety of targeted approaches, such as chemical, chromatographic, molecular, and protein-based techniques, among others, have been frequently used to identify animal species, production methods, provenance, and processing of food products. Although these conventional methods are accurate and reliable, they are destructive, time-consuming, and can only be employed at the laboratory scale. On the contrary, alternative methods based mainly on spectroscopy have emerged in recent years as invaluable tools to overcome most of the limitations associated with traditional measurements. The number of scientific studies reporting on various authenticity issues investigated by vibrational spectroscopy, nuclear magnetic resonance, and fluorescence spectroscopy has increased substantially over the past few years, indicating the tremendous potential of these techniques in the fight against food fraud. It is the aim of the present manuscript to review the state-of-the-art research advances since 2015 regarding the use of analytical methods applied to detect fraud in food products of animal origin, with particular attention paid to spectroscopic measurements coupled with chemometric analysis. The opportunities and challenges surrounding the use of spectroscopic techniques and possible future directions will also be discussed.

## 1. Introduction

In recent years, consumers have become more concerned about the quality and safety of food products and have become keenly interested in knowing more about food authenticity and food fraud. In other words, consumers demand more complete information about their food, including what they are really buying, where the food comes from, and when and how it was produced. Although fraud and adulteration have been practiced since ancient times, it is only in recent years that food authenticity issues have been more exposed, and public attention has been intensively paid to the magnitude of this problem and the serious consequences of food fraud [1,2]. Furthermore, during the current pandemic period with coronavirus raging around the world, affecting every aspect of life, including food choices and nutrition habits, consumers have become even more concerned about safety, accessibility, affordability, and the origin of food products than any time before. This increased interest in food authenticity may also be explained by the numerous food scandals over the last few years (e.g., horsemeat scandal in 2013 and rotten meat from Brazil in 2017) and the increased consumer awareness about the impacts of food fraud in terms of illegal economic gain, as well as negative effects on the public health and the environment. Nonetheless, several recent studies have indicated that fraud or mislabeling is still a widespread practice, especially in food products of animal origin, which are often considered among the most frequently adulterated foods [3,4,5,6]. Market globalization and increases in international trade, driven by fewer obstacles to the export and import of food, a complex food production chain, and the complex nature of food products of animal origin, the huge variety of these products, as well as the emergence of tricky and more sophisticated forms of fraud are some of the reasons that could explain this rise in food fraud and why detection and prevention are challenging tasks [7,8,9,10]. 

**Figure 1:**
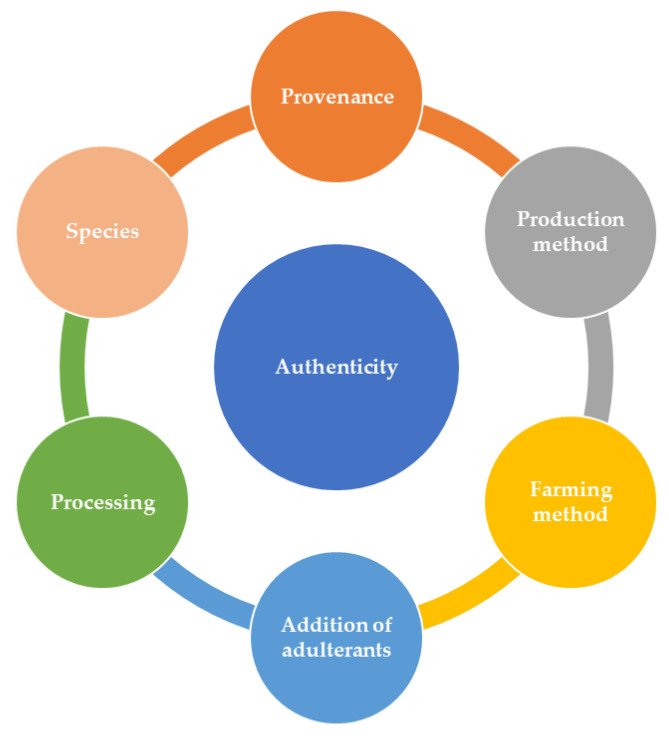
Most reported authenticity issues in food products of animal origin.

**Figure 2:**
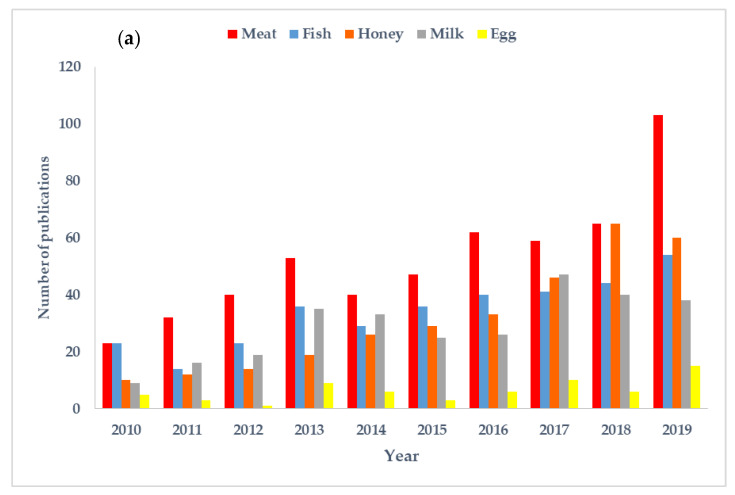

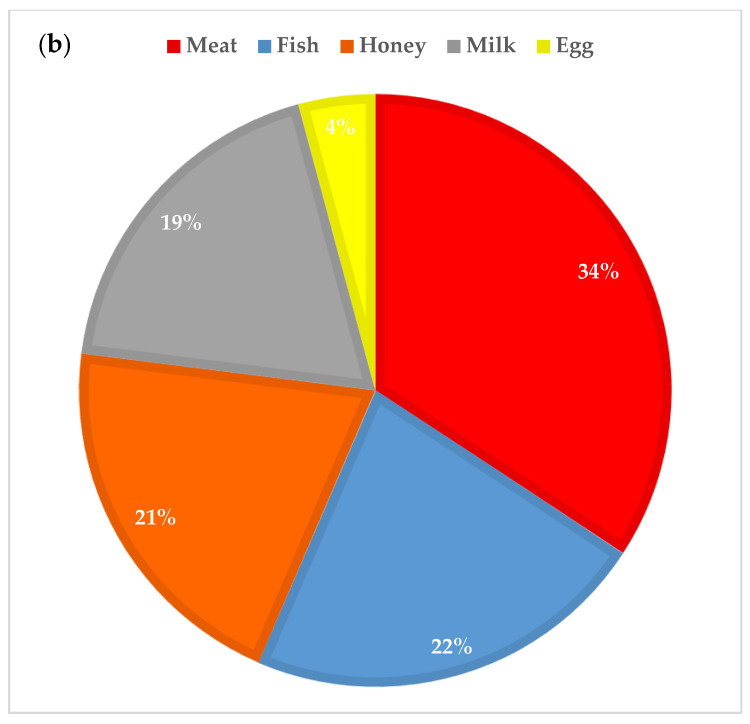
Temporal evolution of published work on the authenticity of different categories of food products of animal origin during the last decade (**a**) and publications distributed between the different food categories (**b**).

Fraud in animal origin products can take many forms, including mislabeling of the provenance (geographical or botanical origin), species substitution, discrepancies in the production method and farming or breading technique, addition of non-declared substances, as well as fraudulent treatments and non-declaration of processes, such as previous freezing, irradiation, and microwave heating (Figure 1). To support this review and obtain the research published in the last few years on the authenticity of food products of animal origin, Scopus database was queried in May 2020, using the keyword “authenticity” or “authentication” and the different categories of animal origin food products. It can be noticed that a huge amount of studies dealing with authenticity and detection of fraud in fish, meat, milk, honey, and eggs has been published in recent years; the number of published works increased from 530 between 2010 and 2014 to 1000 between 2015 and 2019 (Figure 2a). 

Fraud in fish and other seafood is a widespread issue, and seafood products are often ranked among the top food product categories that are susceptible to fraud. Substitution of a high-value fish species with a cheaper alternative and mislabeling of the geographical origin are among the most common fraudulent activities practiced in the fish and seafood sector. Determining whether fish is wild or farmed, tracing farming systems, and differentiating between fresh and frozen–thawed seafoods are among the seafood authenticity topics that have been widely investigated [8]. According to our literature review, meat and meat products are the most studied animal origin foods with regards to authenticity (Figure 2b). Meat authenticity has similar issues to those of fish. To address authentication issues related to muscle foods (fish and meat), a wide range of protein- and DNA-based techniques, chromatography, elemental profiling, and isotopic analysis, among many other measurements, have been frequently applied to this problem [10,11,12]. Similar techniques have also been established in routine analysis for detecting fraud that occurs in other foods of animal origin (e.g., milk and dairy products, honey, and eggs). 

**Figure 3:**
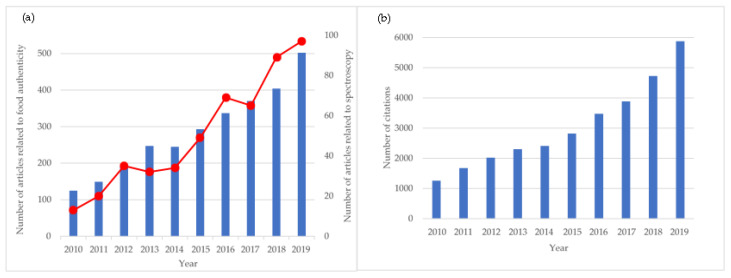
Numbers of published works related to food authenticity (blue bars) and use of spectroscopic techniques in relation to food authenticity (red line) (**a**). Numbers of citations including the words authenticity or authentication and spectroscopy (**b**) since 2010. Data obtained from Scopus database on 25 May 2020.

However, most of the aforementioned analytical methods are associated with several drawbacks, mostly related to the destructive nature of the measurements and the time required to perform the analysis. Therefore, there is still great interest in the development of non-destructive, rapid, accurate, robust, and high-throughput analytical methods for on-site and real-time food authentication. Spectroscopic techniques have gained much importance during the last few years, and spectroscopy has been a popular “buzz word” in the context of fighting fraud and verifying the authenticity of food products. The considerable interest in these non-targeted fingerprinting techniques may be due to the advancements in the analytical instruments and the increasing awareness in the food industry and research on the advantageous aspects of applying such techniques [13]. The number of scientific works regarding the use of spectroscopy for food authenticity increased from 134 papers during 2010–2014 to 369 papers during 2015–2019 (Figure 3a), while the number of total citations (Figure 3b) doubled during the last five years (20,784 citations between 2015 and 2019 versus 9666 citations between 2010 and 2014). Some examples of recent applications of spectroscopic techniques for authentication of food products of animal origin include detection of adulteration in meat [14,15] identification of milk species [16,17], detection of thawed muscle foods [18,19] identification of muscle foods species [20,21,22], and determination of the botanical origin of honey [23,24], among many others. 

Over the last few years, several review papers have been published focusing on either one of the authenticity issues, such as the geographical origin [25,26] or species [27]; or one category of food products of animal origin, such as fish [7,8], meat [28,29], or honey [30]. Other papers have reviewed one specific type of analytical method, such as multielement and stable isotype techniques [11], volatilomics [31], DNA-based methods [32,33], or infrared spectroscopy [34]. The current review will cover the most recent studies that shed light on the various authenticity-related issues (i.e., geographical or botanical origin, species, production method, farming or breeding technique, and processing method) for all food products of animal origin (fish, meat, milk, honey, and egg), highlighting a wide range of both traditional and emerging techniques. This review will first introduce a brief description of the common multivariate data analysis and analytical techniques related to detecting fraud in food products of animal origin. Several examples of applications of conventional and spectroscopic techniques will be then presented, covering the most relevant works published during the last five years. Finally, some difficulties and challenges, as well as future trends in applications of these techniques, will be discussed. To the best of our knowledge, this review paper is the first to combine results from recent studies on a wide range of analytical methods applied to authenticate fish, meat, milk, honey, and egg, as well as their products.

## 2. Multivariate Data Analysis

Traditional chemometric methods are based on linear projections onto a lower dimensional latent variable space, and these powerful and simple methods still dominate the field. However, more flexible and data-intensive machine learning methods have gained traction lately. These methods have the ability to model complex, non-linear relationships; however, the curve fitting procedures, interpretation, and validation are often more complicated. In general, the choice of data analysis strategy depends on the research question, as well as the type and size of the available data. 

The data analysis pipeline consists of preprocessing, data exploration, modeling, and validation. The following sections give a brief description of each of these steps, with the main emphasis on recent trends and developments. For detailed overviews of data analysis in food authenticity, please refer to [35,36,37,38].

### 2.1. Data Preprocessing

The aim of preprocessing is to reduce non-relevant variations in the signal stemming from instrumental artifacts, surrounding effects, or sample preparation. The most used methods include standard normal variate (SNV), (extended) multiplicative signal correction ((E)MSC), derivatives, smoothing, baseline corrections, and peak alignments, which are often used in combination. The choice of preprocessing method is critical for the subsequent modeling and interpretation [39,40], and should be chosen based on knowledge of the samples and the measurement platform. Recent research suggests various strategies for making the modeling less sensitive to preprocessing, for instance by using a boosting approach [41], through Tikhonov regularization [42], or by using convolutional neural networks [43,44,45]. 

### 2.2. Data Exploration

Data exploration is an important step prior to the actual modeling. The aim is to gain an overview of the data, deal with outliers, evaluate the effects of preprocessing, and get a first impression of the potential for discriminating between samples. Principal component analysis (PCA) is the most used tool for data exploration, providing a linear transformation of the original data by maximizing the explained variance. Cluster analysis is another group of exploratory methods based on a certain distance or similarity measure between samples. These methods can be more flexible than PCA, depending on the chosen similarity metric, and may be useful for very large sample sizes. 

### 2.3. Modeling

Authentication tasks mainly aim to determine which category a food item belongs to, i.e., classification. There are two main approaches to classification: class modeling and class discrimination [46,47,48]. While class modeling focuses on modeling the similarities among samples from the same category, class discrimination focuses on finding the differences between a set of predefined categories. The most used methods in the scientific literature are the soft independent modeling of class analogies (SIMCA) and partial least squares discriminant analysis (PLS-DA) classical chemometric methods for class modeling and discrimination, respectively; however, methods such as support vector machines (SVM), random forests (RF), k-nearest neighbor (k-NN), and different types of neural networks (NN) are also frequently applied. Quantitative prediction models are also relevant in some cases, for instance when the objective is to quantify the amounts of specific adulterants. An overview of alternative methods for class modeling, discriminant analysis, and quantitative prediction can be found in [35,36,37,38]. 

*Data Fusion*: Data or sensor fusion is an emerging topic within food authentication. A combination of several instrumental techniques can lead to more accurate results, either by providing complementary information or by reducing uncertainty [49,50,51,52,53]. Data fusion is also an active research area in fields other than authenticity, and new methods for explorative analysis, classification, and prediction are presented frequently. In principle, all multivariate methods can be used for data fusion by (1) combining all the measured variables directly, called low-level data fusion; (2) combining extracted features such as principal components, called mid- or feature-level data fusion; or (3) combining predictions or classifications from different techniques through voting, called high- or decision-level fusion. There are also several methods that are tailored for data fusion problems. Examples of newly developed explorative techniques include methods that separate common and distinctive variations in multiple data blocks [54,55], whereas sequentially orthogonalized PLS (SO-PLS) [56,57] is a common example of multiblock regression methods. 

*From Small to Big Data*: In general, the traditional chemometric methods, such as PCA, SIMCA, and PLS-DA/PLSR, are suited for small feasibility studies, while larger studies allow for use of more data-intensive methods, such as SVM, RF, and NN. In industrial applications, however, databases with hundreds of thousands of samples are often available. Such huge data sets call for completely different data analysis strategies. There has so far been little focus on authentication models based on large databases in the scientific literature, mainly because these databases are not open. There are, however, a few exceptions showing that local modeling is a promising strategy [58,59]. In local modeling, a new model is fitted for each new sample to be predicted, using only a subset of spectrally similar samples as a calibration set. More research is needed on the use of local modeling for classification and on the analysis of large databases in general.

### 2.4. Validation

One of the main barriers for the successful implementation of fingerprinting techniques in food authenticity is the lack of proper validation schemes [2,60,61,62]. A full validation scheme consists of four phases: (1) optimization of the analytical procedure, (2) statistical model selection and parameter optimization, (3) testing of the model performance, and (4) stability testing by system challenges [60]. Most published feasibility studies stop at phase two or three, while phase four is essential for successful implementation. 

Phase one is specific for the analytical technique and will not be covered here. The aim of phase two is to select an optimal modeling strategy and model parameters. This is usually done by resampling methods, such as cross-validation or bootstrapping. Phase three involves testing the model performance using an independent test set, while phase four tests the extrapolation of the model, e.g., overtime or for different instruments and locations. Thorough reviews of both numerical and conceptual aspects of validation are given in [63,64]. 

## 3. Overview of Fraud Detection Techniques

### 3.1. Spectroscopic Techniques

#### 3.1.1. Vibrational Spectroscopy

Innovation pathways in vibrational spectroscopy during this past half decade are preludes to potential impacts and further practical achievements in the next half decade. Vibrational spectroscopy techniques, including infrared spectroscopy in the near (NIR)- and mid (MIR)-infrared spectral ranges, as well as Raman spectroscopy, enable a fingerprinting chemical analysis of an intact food sample in situ for adulteration in real time. The sample remains intact for confirmatory analysis using other techniques. Spectroscopic technologies require high levels of rigor in the evidence for authentication of both the food or food product and of the adulterant or contaminant.

Variance in the spectral signature of the food always can complicate the capacity to distinguish the amount and composition of an adulterant or contaminant. Recent state-of-the-art authentication of milk products has been reported [65,66]. The authentication of raw milk involves a different process—knowing its fingerprint identity enables detecting adulteration [67]. Products made from milk have also been authenticated. Desi ghee made from buffalo and from cow milk can be differentiated [68], while butter containing lard [69] and cream and yogurt [70] can be distinguished with chemometrics.

Authentication in meats is required for foods that are labeled as individual meats [71]. Horsemeat in minced beef [72], beef and mutton in pork [73], and rainbow trout in Atlantic salmon [74] each require sufficient data specific to substances to be labeled to assure the meat contaminant is properly characterized in order to identify markers characteristic of each additional component. Spectral data on the primary meat preferentially needs to be oversampled relative to that of a contaminant, or of minor or occasional components that could be misinterpreted as unrelated to the original meat. Factors such as diet can alter vibrational fingerprints. Eggs from poultry fed omega-3 fatty acids contain an intentional adulterant that can be detected in the spectral signature of the eggs [75]. Work involving fish fillet authentication using vibrational spectroscopy has also been published [21,76].

#### 3.1.2. Nuclear Magnetic Resonance

Nuclear magnetic resonance (NMR) spectroscopy, despite being a very well-established methodology in food analysis, has had limited new publications over the last five years. The major difficulties are that foods are inherently mixtures of components and adulterants may or may not be mixtures. Thus, identifying NMR chemical shifts that do not belong in a particular food first requires authentication of the fact that a particular set of peaks may not arise occasionally (i.e., more rarely) on its own. This is an innately complicated process because one needs to ascertain which chemical shifts are correlated. A major advantage of NMR is that modern NMR techniques can trace the fingerprints from finger to finger and ascertain one part of a fingerprint belongs to another hand. Publishing the results of such an effort is often difficult because someone else may have found the same compound in this (or another) food product. Further, if the compound found is of little apparent biological or food property relevance, journal reviewers can deem such research as having a correspondingly low relevance. 

The specificity of NMR complicates the authentication of the composition of an adulterant. A unique and specific NMR peak at best detects only a single component of an adulterant. If an adulterant happens to be a mixture of components, NMR is useful only for detecting chemical components one at a time. Thus, if in minced meat labelled beef porcine fat can be detected as an adulterant, NMR can only identify a chemical shift, which identifies a site on a specific unsaturated lipid as foreign to beef. It cannot likely identify which animal (or plant for that matter) was the source of the product contamination. Once markers have been authenticated properly to a specific chemical structure, this fingerprint is treated as a positive result awaiting verification by some other technique. Verifying the food commodity that has been used for adulteration requires significantly more spectroscopic data. Each and every spectral identification result that can be detected in a specific matrix can be a significant challenge and are important to know. Publishing such a finding is a more complicated endeavor.

Three recent NMR manuscripts involved detection of adulteration in milk, powdered milk, or butter [77,78,79]. Two publications involving edible lipids, including milk, additionally used more complicated NMR experiments (time-domain NMR and ^13^C inept NMR) [80,81]. The more complicated NMR techniques enhance the resolution and quality of data collected. A similar enhancement using improved technologies and methodologies in milk was reported using Raman chemical imaging techniques [82]. One manuscript reported on the authentication of krill oil using NMR techniques [83]. 

The focus on NMR research in authenticating lipid compositions in foods is because specific lipids in mixtures of lipids appear to be characteristic of their origin. The high resolution of NMR enables deconvolution of the specificity of the lipid composition at the molecular level. Solvent effects, however, appear to complicate spectral assignments. Two publications verified that NMR techniques can fully distinguish omega-3 from omega-6 fatty acids in mixtures [84] and among three omega-3 fatty acid structural analogs, each in an intact lipid environment [85]. Authenticating the fingerprints of lipids is an essential component of and prerequisite for verifying adulteration correctly.

#### 3.1.3. Fluorescence Spectroscopy 

Fluorescence spectroscopy is based on measurement of the spectral distribution of the intensity of the light emitted by electronically excited molecules. Fluorescence coupled with chemometrics has been widely used in food studies, including for products of animal origin [86,87,88,89,90,91]. The main advantages of fluorescence as compared to other spectroscopic techniques are its higher sensitivity and selectivity. Due to these features, fluorescence is particularly useful for studying minor and trace components in complex food matrices [87,91]. Characterization of real multifluorophoric food samples requires more advanced measurement techniques than conventional emission or excitation spectra. The advanced fluorescence techniques have often been used in food studies, including excitation–emission matrix (EEM) fluorescence spectroscopy, synchronous fluorescence spectroscopy (SFS), and total synchronous fluorescence spectroscopy (TSFS) [87,92]. 

Fluorescence patterns of food products are usually complex. Fluorophores in food include natural food components, process-derived compounds, food additives, and contaminants [89]. Autofluorescence of meat and fish originates mainly from collagen, adipose tissues, proteins, and oxidation products [89]. Milk and dairy products contain several intrinsic fluorophores, including free aromatic amino acids, nucleic acids, aromatic amino acids in proteins, vitamins A and B_2_, nicotinamide adenine dinucleotide (NAD), chlorophyll, and oxidation and Maillard reaction products [86,90]. Fluorescence in honey is ascribed to proteins, polyphenolic compounds, and Maillard reaction products [23,93,94]. The unique fluorescence patterns of food products have been successfully utilized in authentication studies of food of animal origin, including meat [95,96], fish [97,98], milk [16,17] dairy products [86,90], and honey [23,88,99,100,101,102].

#### 3.1.4. Other Spectroscopic Techniques

The number of studies on the potential use of novel spectroscopic techniques to detect fraudulent practices encountered in the food chain has gradually increased in recent years. In this section, information is given on the applications of laser-induced breakdown spectroscopy (LIBS), terahertz (THz) spectroscopy, and hyperspectral imaging (I) in food adulteration analysis.

LIBS has been presented as a potential alternative to the existing analytical atomic spectrometry techniques used to determine the elemental composition of food. Most of the samples need a minimum or no sample preparation to be analyzed by using LIBS. The simultaneous analysis of multiple elements can be achieved. It is highly applicable to at-, on-, and in-line measurements and remote sensing, enhancing its potential as an analytical technology process [103,104]. LIBS coupled with several chemometric methods has been widely used for species discrimination [105], determination of adulteration [106], and spatial mapping of the sample surfaces in meat, milk, and other products [107,108]. Recently, some studies utilized LIBS for analysis of honey adulteration [109,110] and determination of its geographical origin [111,112]. Although there is a significant amount of research in the literature reporting the high potential of LIBS as an at-line monitoring tool for the industry, there is still a need for further improvements in system components and configurations. Besides, more research is required to recommend alternatives to reduce the matrix effect and minimize sample preparation procedures in order to improve the predictive accuracy. Peng et al. have described the significant challenges and possible solutions to these in order to speed up the use of LIBS as an in situ monitoring tool [113,114]. 

Terahertz spectroscopy (THz) is another technique that provides an excellent alternative to X-rays in order to obtain high-resolution images from the interiors of solid objects. Frequency-domain and time-domain measurements are performed for both imaging and spectroscopy with THz waves [115]. There are a limited number of studies on the use of THz spectroscopy for the determination of food adulteration, which were previously compiled by Afsah-Hejri [115] and He [116]. Adulteration of milk with a fat powder [117], discrimination of honey samples [118], and determination of honey adulteration [119] were some of the recent study topics. 

Hyperspectral imaging (HSI) is another relatively new technology, which has explicit potential to satisfy the needs for remote and real-time monitoring techniques. Being rapid, non-invasive, and providing spectral and spatial features simultaneously are some of its significant advantages. Numerous articles describe the pros and cons of HSI-based methods for food authenticity and adulteration analyses [14,15]. Nowadays, low-cost, rapid, and simple multispectral imaging systems are being designed for the determination of particular adulterations [120]. Efforts are being made to offer alternative methods for the interpretation of HSI data. The transition from the use of linear classifiers to machine learning and deep learning solutions offers a great variety of opportunities [121]. Another trend is to employ miniature devices called single shot or snapshot hyperspectral sensors, which are ultra-portable and able to acquire data at video rates [122]. The enormous potential of the HSI technique to detect many aspects of food adulterations has been shown in the literature. Even so, enhancement of the spectral and spatial resolution and presentation of alternative technologies for advanced data analysis would be positive contributions to the accuracy and cost-effectiveness of the developed methods.

### 3.2. Other Analytical Methods

#### 3.2.1. DNA-Based Techniques

To date, many DNA-based detection methods have been developed to determine animal species in food products. In particular, DNA-based methods have been used to detect target species in processed foods, because DNA is stable at high temperatures and pressures. Sequencing-based techniques (such as DNA barcoding and minibarcoding), polymerase chain reaction (PCR) coupled with restriction fragment length polymorphism (PCR-RFLP), real-time PCR, multiplex PCR, and species-specific PCR are among the most used techniques [32,123,124].

Identification of short DNA sequences, called DNA barcodes, has been widely exploited for species discrimination. DNA barcoding and minibarcoding were used to authenticate animal-derived food products sold in the Chinese market [125] and to identify selected brands of locally-produced canned and dried sardines in the Philippines [126]. 

In PCR-RFLP, the PCR products are cleaved with restriction enzymes, followed by gel electrophoresis and blotting [32,123]. The technique was successfully applied to differentiate four commercial shrimp types in India, and the developed PCR-RFLP protocol was validated by analyzing 52 commercial shrimp products [127].

Real-time PCR and multiplex PCR methods are the most common detection technologies in meat and meat products, fish and seafood, and other food categories that are known to have a high incidence of adulteration [124,128,129,130]. There are numerous reports in the literature demonstrating that real-time PCR is a powerful method that can be used as a reliable and sensitive technique for meat identification. For example, in one of the recent studies, a real-time PCR assay was developed for the detection of raw donkey meat and different processed meat mixtures [131]. Fang and Zhang used real-time PCR and TaqMan-specific probes for the detection of murine components in mutton meat products [129]. The results showed that the limit of detection was lower than 1 pg of DNA per reaction and 0.1% murine contamination in meat mixtures.

Many researchers have applied multiplex PCR methods for identification of meat species for simultaneous and rapid detection of multiple species in a single reaction. For example, two direct-triplex real-time PCR systems based on intercalating dyes were applied as a robust and precise quantitative PCR assay for meat species identification [124]. No DNA extraction was required and 92.5% of market samples of six commonly eaten meat species were successfully amplified. The multiplex PCR method was also applied to detect chicken, duck, and goose in beef, mutton, pork, or quail meat samples [132]. In a similar study, a multiplex PCR assay was used to identify lamb, beef, and duck in a meat mixture before and after heat treatment [133]. Similar approaches were developed to monitor commercial jerky products [134]; to detect chicken and pigeon in raw and heat-treated meats [135]; and to detect chicken, turkey, and duck in processed meat products [130]. Recently, a fast multiplex real-time PCR with TaqMan probes was performed to simultaneously detect pork, chicken, and beef in processed meat samples [136].

The species-specific PCR method has been used to a great extent for meat species identification in foods because of its high specificity and rapidity. For instance, El-Razik and co-authors used a species-specific PCR test to differentiate donkey and horse tissue in cooked beef meat products in Egypt [137]. In another study, a species-specific PCR was developed for the identification of beef in India [138].

In addition, more advanced high-throughput DNA sequencing methods, such as next-generation sequencing (NGS) [139,140], have emerged in recent years as valuable techniques for carrying out untargeted screening of complex samples.

#### 3.2.2. Protein-Based Techniques and Related Methods

Chromatographic, electrophoretic, and immunological methods have been widely used for different authenticity issues for food products of animal origin [29,123,141,142]. Different mass spectrometry (MS) techniques have emerged in recent years, and along with chromatographic and NMR techniques have become some of the most commonly applied approaches for metabolomic fingerprinting [142,143]. Traditionally, MS methods are coupled with chromatographic separation techniques, such as liquid chromatography mass spectrometry (LC-MS) [142]. More recently, direct MS analysis approaches, such as matrix-assisted laser desorption/ionization-time of flight (MALDI-TOF), real-time techniques (e.g., direct analysis in real-time (DART) technique), and high-resolution mass spectrometry (HR-MS), among others, have been developed and applied to many authentication problems [123,144,145,146,147]. For example, a DART HR-MS method was developed to discriminate Canadian wild salmon from the farmed fish produced in Canada, Chile, and Norway [144]. The results showed that PCA applied to the 30 most abundant signals generated from fatty acids after the DART HR-MS analysis of fillet lipid extracts enabled a rapid discrimination between farmed and wild fish, whereas discriminant analysis (DA) gave a correct classification rate of 100%. In another study, the differences between rainbow trout and king and Atlantic salmons were studied using a lipidomical method based on hydrophilic interaction chromatography MS [147]. PCA was applied to recognize the variance among these fish species, which was attributed to the genetic origin, living environment, and feed ingredients, among others. A novel method based on quadrupole time-of-flight (Q-TOF) MS coupled with a surgical diathermy device was recently developed to distinguish cod from oilfish in real time [145]. PCA demonstrated that the clusters of oilfish were well separated from those of cod, while the application of discriminant analysis models showed that the fish tissue can be authenticated with 96–100% accuracy. Another recent study investigated the potential of ultra-performance liquid chromatography–triple time-of-flight–tandem mass spectrometry (UPLC−triple TOF−MS/MS) to determine lipid composition in the muscle tissue of four popularly consumed shrimp species [146]. About 600 lipid compounds from 14 classes were characterized and quantified, and PCA results of lipid profiles allowed the different species to be distinguished. In a similar investigation, the use of LC-TOF−MS allowed the detection of commercially available, highly processed mixed-meat products, including duck, goose, and chicken, along with pork and beef [148].

Besides the chromatographic and mass spectrometry techniques, enzyme-linked immunosorbent assay (ELISA) is one of the most widely used methods for meat identification, because it is cheap and easy to perform [141,149].

Although the aforementioned techniques have several advantages, such as stability during thermal processing and high sensitivity and selectivity, most of these measurements are time-consuming because several steps are required for sample preparation, protein extraction, and lipid extraction. In addition, the technical difficulty with MS and PCR in food adulteration is that they are useful mainly and sometimes only after the rest of the chemistry and spectroscopy work has been completed. Such techniques are especially valuable for verifying adulterations detected in situ by other technologies.

#### 3.2.3. Isotopic Technique

As the isotopic compositions of the plants or animals reflect the condition of natural environment where they grew up, the light stable isotopes ^13^C/^12^C, ^18^O/^16^O, ^2^H/^1^H, and ^15^N/^14^N, ^34^S/^32^S; and the heavy isotopes ^11^B/^10^B and ^87^Sr/^86^Sr are commonly used in food authentication [11]. Preliminary studies have demonstrated the usefulness of stable isotope analysis in determining the origins of animal origin products [150,151,152,153,154]. However, the animal origin products had more complicated life cycles than the plant origin products. The stable isotopes such as δ^2^H and δ^18^O were more likely to be affected by the ambient environment [151,155]. Camin and co-authors [151] reported the H/O ratios of Italian rainbow trout fillets were positively interrelated with the O ratio of tank water. However, the other stable isotopes ^13^C/^12^C, ^15^N/^14^N, and ^34^S/^32^S were reported to be affected by diet [11,156,157]. Taking shrimp as an example, the δ^13^C and δ^15^N values in shrimp are significantly related to the food sources [158]. During shrimp culture, the farmers may use several brands of commercial feeds with different ingredients and isotopic signatures. Li et al. [156] reported that the δ^13^C and δ^15^N values in 16 commercial feeds used in shrimp culture in China ranged from −23.03 to −24.75‰, and from 2.1 to 8.18‰, respectively. The dietary shifts could influence the stable isotope signature of shrimp. The effects of diet on the stable isotope signature of animal origin products should be considered when using traceability methods. Moreover, animals can only be sampled for traceability purposes when they are in isotopic equilibrium with their diet. In a recent study, Li and others suggested the sampling of shrimp that have been consistently fed with the same feed for more than twenty days [158].

The stable isotopes of animal origin products could also be affected by other environmental factors, such as culture seasons and salinity [159,160]. Compared with the marine ecosystem, the freshwater ecosystem generally has low δ^13^C and δ^15^N values [159,161]. Previous studies reported different δ^13^C and δ^15^N values in shrimp and fish cultured in freshwater and seawater [156,159]. Hence, all of these factors should be compared when using isotopic traceability methods to allow for deter animal origin product fraud.

#### 3.2.4. Elemental Technique

The isotopic technique is usually combined with an elemental profiling technique to increase the accuracy of the traceability methods [157,159,162,163,164,165]. Elemental profiling techniques rely on digestion of samples into ions, then concentration of the ions is followed by spectroscopic analysis, including atomic absorption spectroscopy (AAS), inductively coupled plasma–optical emission spectroscopy (ICP-OES), and ICP–mass spectrometry (ICP–MS) [11]. The analyzed elements include K, Ca, Na, Mg, Cu, Fe, Mn, Al, Zn, As, Cd, Cr, Mn, Ni, Zn, Ba, Sr, Li, Se, Co, Ti, and V. Those elements include bulk structural elements (P, S, Si, etc.), macroelements (K, Ca, Na, Mg, etc.), trace elements (Cu, Fe, Zn etc.), and ultratrace elements (As, Cd, Cr, Mn, Ni, Co, V, etc.). Both non-metal elements (P, S, As, etc.) and metals (Mn, Fe, Cu, etc.) have been used in analysis [166]. In recent years, the rare earth elements (REEs), including Y, Ce, Nd, Pr, Sm, Er, and Eu, have also been used in traceability methods [159,167]. Databases generated by chemical analysis are subjected to multivariate analysis for data exploration.

Elemental profiling was used in geographic traceability testing of plant origin products, because element compositions of the specimen were believed to be a distorted reflection of the elemental profiling of the soil environment in which they grew [11]. This fact is more complicated for animals, who derive their elements not only from the environment but also the food they consume. Hence, feed is a factor that needs to be seriously considered in the traceability of animal origin food products. Mineral concentrations of feed, such as fish feed, vary greatly due to differences in raw ingredients, addition of specific macro or trace mineral premixes, or contamination [11]. The culture environment of animals is also more complicated than plants and the elemental profiling of animals can be affected by factors such as the culture season, size of the animal, species, and water quality [160,168,169]. For example, Han and others [168] reported that the element compositions of salmonid obtained from the reservoir were vulnerable to seasonal changes. Although studies have demonstrated the usefulness of elemental profiling in tracing the origin of animal origin products, all factors should be considered in future studies to strengthen the accuracy of the method.

## 4. Examples of Recent Use of Spectroscopic and Traditional Methods to Detect Fraud 

### 4.1. Fish and Seafood Products

*Identification of Geographical Origin*: Provenance or geographical origin has become one of the most important authenticity issues for fish and seafood due to the increasing awareness among consumers of the impacts of their purchasing choice of seafood on the marine environment. Many consumers are becoming more worried about fraud, which occurs when fraudsters conceal the geographical origin or hide an illegally harvested protected species or a species from a protected area. Thus, reporting of the country of origin or place of provenance of seafood is essential in the fight to preserve sustainable fisheries, for better management of fish stocks, and to prevent unreported and unregulated fishing. This is why a requirement with respect to a clear indication of the geographical origin of seafood products has been implemented in many countries, such as the European Union and the USA [123,170]. 

Several analytical methods have been developed in order to identify the origin of seafood. Trace elements fingerprinting, stable isotope analysis, and DNA-based methods are among the most used approaches for this purpose. While these techniques show promise for definitively identifying the geographical origin of fish and other seafood [32,171,172,173], they have certain drawbacks, especially in terms of the required time and the destructive nature of measurements. 

Recently, some studies have demonstrated the usefulness of spectroscopic techniques for monitoring the geographical origin of seafood [174,175,176] (Table 1). In one of these studies, NIR spectroscopy was applied to classify tilapia fillets according to their 4 geographical origins, namely Guangdong, Hainan, Guangxi, and Fujian in China [174]. SIMCA performed on the spectra showed a classification ability ranging from 75% for the Guangxi provenance to more than 80% for the other origins. In another study, a better classification efficiency of sea cucumber originating from nine Chinese locations was obtained using NIR spectroscopy combined with PCA [175]. More recently, a similar technique was used to trace the geographical origin of European sea bass collected from the Western, Central, or Eastern Mediterranean Sea [176]. Results showed correct classification rates of 100% 88%, and 85% for the fish originating from the Eastern, Central, and Western Mediterranean Seas, respectively, with lipid absorption bands being the major contributor to the discrimination ability of the spectra. 

In the literature, there are few studies regarding the use of NMR or fluorescence spectroscopy for monitoring of the geographical origin of seafood. In one of these scarce studies [177], ^1^H NMR spectroscopy combined with SIMCA and PLS-DA was successfully applied to discriminate caviar cans originating from producers in the Aquitaine region in France from other producers. Therefore, more spectroscopic studies should be conducted on this topic in order to draw valid conclusions about the potential of these techniques for determining the geographical origin of fish and other seafood. 

*Tracing Wild and Farmed Seafood and Farming Methods*: During the last few years, there has been a rapid expansion of aquaculture as a result of overfishing and decreasing wild fish stocks. Consumers generally prefer wild fish over farmed fish, and when it comes to farming, organically farmed fish is usually believed to be healthier and of higher quality in terms of animal welfare and environmental perspectives compared to conventionally farmed fish. This is why labeling farmed fish as wild or conventionally raised fish as organic is considered a fraudulent practice. 

Various approaches have been proposed over the years to trace production methods and farming systems. Elemental profiling, stable isotopes, fatty acid analysis, or combinations of these methods have been extensively applied [144,173,178,179]. For example, a technique based on stable isotope analysis allowed differentiation of organically farmed from conventionally farmed salmon and brown trout, independent of the type of processing, i.e., raw, smoked, or graved [180]. In another study, the combination of stable isotope ratio analysis with multielement analysis gave a correct classification of 100% of shrimp samples according to their geographical origin and production method (i.e., wild or farmed), while 93.5% of the samples were correctly classified according to species [163]. A more recent study confirmed the positive effects of combining the stable isotopes and elemental profiling techniques to determine the origin and production method of Asian sea bass collected from Australian and Asian sources [160]. 

Only a few studies regarding the use of spectroscopic techniques for distinguishing between wild and farmed fish or between different farming regimes have been published so far. Xu and co-authors studied the possibility of discriminating wild and farmed salmon with different geographical origins and farming systems using HSI operating in two spectral ranges (spectral set I: 400–1000 nm; spectral set II: 897–1753 nm) coupled with different chemometric tools [181]. The best results were obtained with SVM applied to spectral set I, giving a correct classification rate of 98.2%. In a more recent study, NIR spectroscopy in the range of 1100–2500 nm was applied to authenticate European sea bass [176]. Slight separation was observed between fish groups when PCA was applied. However, PLS-DA allowed a clear discrimination between wild and farmed fish with a correct classification rate of 100% being achieved. Moreover, the different farming systems, including extensive, semi-intensive, and intensive farming, were discriminated from each other with correct classification rates of 67%, 80%, and 100%, respectively. In this study, the absorption bands of proteins were reported to be the greatest contributors to the discrimination ability of the spectra. 

*Detection of Species Fraud*: Substitution of valuable marine species with less desirable or cheaper ones is the most common type of fraud in fish and other seafood. Detection of this type of fraud is difficult, especially if the fish is in the form of a fillet without skin or if the seafood product has been processed [182,183]. Given the widespread practice of species fraud and the serious consequences it can have, it is no wonder that a wide variety of conventional methods and spectral fingerprinting techniques have been investigated in order to aid in addressing this issue. DNA analysis and MS methods are among the most commonly used techniques in this regard [126,145,146,184,185].

**Table 1:** Examples of applications of spectroscopic techniques with respect to various authenticity issues for fish and other seafood.

Fish or Other Seafood	Authenticity Issue	Analytical Technique	Modeling Method	Reference
Horse mackerel, European anchovy, red mullet, bluefish, Atlantic salmon, and flying gurnard	Species identification/detection of thawed fish	Raman	PCA	[186]
Pacific white shrimp	Origin authentication	NIR HSI (874–1734 nm)	PLS-DA, LS-SVM, ELM	[187]
Norwegian salmon	Species identification	FT-IR (4000–450 cm^−1^)	PLS-DA	[188]
Carotenoid, salmonid/freshwater, saltwater fishes	Species identification/origin	Raman	HCA	[189]
Seven freshwater fish species	Species identification	NIR (1000–1799 nm)	PCA, LDA	[190]
Fish surimi; white croaker, hairtail, red coat	Species identification	FT-IR (2500–25000 nm)	PCR	[191]
Fish surimi; white croaker, hairtail, red coat	Species identification	NIR (1000–2500 nm)	DA	[192]
Freshwater shrimps	Addition adulterant	LF-NMR, MRI	PCA, PLSR	[193]
Tilapia	Detection of thawed fish	NIR (1000–2500 nm)	PCA	[194]
Crucian carp	Detection of thawed fish	VIS/NIR HSI (400–1000 nm)	PLS-DA	[195]
Grass carp	Detection of thawed fish	VIS/NIR HIS (400–1000 nm)	SIMCA, PLS-DA, LS-SVM, and PNN	[196]
Shelled shrimp	Detection of thawed products	VIS/NIR HSI (400–1000 nm)	SIMCA, RF	[197]

PCA, Principal Component Analysis; PCR, Principle Component Regression; LDA, Linear Discriminant Analysis; DA, Discriminant Analysis; RF, Random Forest; SIMCA, Soft Independent Modeling of Class Analogy; PLS-DA, Partial Least Squares Discriminant Analysis; PLSR, Partial Least Squares Regression; LS-SVM, Least Squares Support Vector Machines; PNN, Probabilistic Neural Network; VIS/NIR; Visible–Near-Infrared Spectroscopy; his, Hyper Spectral Imaging; LF-NMR, Low-Field Nuclear Magnetic Resonance; MRI, Magnetic Resonance Imaging; FT-IR, Fourier-Transform Infrared Spectroscopy; ELM, Extreme Learning Machine; HCA, Hierarchical Cluster Analysis.

Several spectroscopic techniques in conjunction with chemometric tools have been used to identify fish species and detect fraud. Alamprese and Casiraghi used FT-NIR and FT-MIR data coupled with two classification techniques (i.e., SIMCA and linear discriminant analyses (LDA)) in order to discriminate valuable fish species (i.e., red mullet and plaice) substituted with cheaper ones, namely Atlantic mullet and flounder [76]. The best results were obtained by the LDA model, giving a 100% correct classification rate for red mullet and Atlantic mullet, regardless of the used spectroscopic techniques. Regarding discrimination between plaice and flounder species, the best results were obtained using FT-IR, with more than 83% prediction ability and 100% specificity being achieved. 

The progress in miniaturization accompanied by software development has led to the emergence of several handheld and portable devices based on spectroscopy for many applications in the food industry [198,199]. In this respect, an investigation based on a handheld NIR device and FT-NIR benchtop spectrometer was carried out in order to discriminate Atlantic cod from haddock fillets and patties [200]. The results obtained by applying LDA and SIMCA models to the spectra using the portable device demonstrated an equivalent discrimination power to those obtained by the stationary benchtop instrument. 

Besides NIR spectroscopy, other vibrational spectroscopic techniques have been widely employed to detect fraud in seafood species. For instance, MIR spectroscopy was applied to detect fraud involving substituting Atlantic salmon with rainbow trout in mini-burgers [201]. Using PCA, the authors succeeded in discriminating 11 formulations with different percentages of these two species, and the percentage of the fraud in the mixture was successfully predicted using PLSR. The same authenticity issue (i.e., species identification) was later studied in a similar investigation, but with a different vibrational spectroscopic technique, namely Raman spectroscopy [74].

Again, few or no studies have been found in the literature regarding the application of NMR or fluorescence spectroscopy. A recent study investigated the use of HSI in 4 different spectroscopic modes, including reflectance in the VIS/NIR region, fluorescence, reflectance in the short-wave infrared region, and Raman spectroscopy for discriminating between 6 fish species and differentiating between fresh and frozen–thawed fish [21]. By testing several machine learning classifiers, the authors obtained the best results when using the VIS/NIR and the short-wave infrared techniques for the identification of fish species and detection of thawed fish, respectively. 

*Checking of Processing Treatments*: Fish and other seafood products are highly perishable foods that must be processed or preserved properly and rapidly after catch or harvest in order to extend their shelf life and maintain quality. Freezing has been widely applied as one of the most common ways of achieving this purpose. However, fresh products are often considered by consumers to be of superior quality and are usually sold at higher prices then frozen food. Therefore, discrimination between fresh and frozen products is one of the most important authenticity issues. Enzymatic, electrophoretic, and histological methods have been commonly used to detect thawed fish and seafood [202,203,204,205]. 

Vibrational spectroscopy, NMR, and fluorescence spectroscopy have shown considerable potential as interesting alternatives to traditional measurements used to differentiate fresh from frozen–thawed seafood. For example, differentiation of fresh and frozen–thawed Atlantic mullet fillets was successfully reported with the use of SIMCA applied to FT-IR, with values of more than 98%, 88%, and 95% being obtained for classification ability, prediction ability, and specificity, respectively [76]. Similar results were reported by using PLS-DA on VIS/NIR spectra obtained for fresh and frozen–thawed tuna, and high sensitivity, specificity, and accuracy of the model were achieved [206]. 

Unlike the other vibrational spectroscopy, very little work has been devoted to examining the potential of Raman spectroscopy to differentiate fresh and frozen–thawed fish. Fat extracted from six fish species, namely horse mackerel, European anchovy, red mullet, bluefish, Atlantic salmon, and flying gurnard, was analyzed using Raman spectroscopy in order to discriminate between fresh, once-frozen–thawed, and twice-frozen–thawed fish [186]. PCA models were developed and displayed a clear discrimination between the 3 states of each fish species, indicating a strong ability of this technique to rapidly detect changes in the lipid structures of fish species compared to gas chromatography, which is usually used in classical analysis. 

Although NMR has been widely used to monitor changes in fish occurring during freezing and frozen storage [207], little work has been done regarding the use of this technique to differentiate between fresh and frozen–thawed fish. Recently, NMR was used to deal with freshness authentication of Atlantic salmon by analyzing metabolic changes that occur during the thawing process [19]. A PCA score plot showed distinct fresh and frozen–thawed groupings, while the discrimination ability was attributed to the formation of aspartate in the thawed salmon. 

Few studies on fluorescence spectroscopy have been reported in the scientific literature, showing the possibility of the application of this technique to study different authenticity issues in seafood. For instance, the potential of front-face fluorescence spectroscopy was investigated to discriminate between fresh and frozen–thawed sea bass [208]. In this study, four fluorophores were examined, including NADH (excitation at 340 nm), tryptophan (excitation at 290 nm) riboflavin (excitation at 380 nm), and vitamin A (emission set at 410 nm). The results showed that this technique coupled with some appropriate chemometric tools was able to discriminate not only between fresh and frozen–thawed fish, but also between frozen fish of differing quality before freezing and storage.

Many studies have demonstrated the potential use of HSI for various authentication purposes [209]. Discrimination between fresh and frozen–thawed cod fillets was investigated by using VIS/NIR HSI adapted for online measurements of fish fillets moving on a conveyor belt at a speed of 40 cm/s, a rate that meets the industrial production requirements [210]. The results showed that the technique was able to differentiate between both fresh and frozen–thawed cod fillets and between the fillets according to different freezing and thawing protocols as a function of sample freeze–thaw history. In this study, the discrimination ability was attributed to variations in the visible region of the spectrum induced by oxidation of hemoglobin and myoglobin and to scattering changes caused by protein denaturation and other structural modifications during the freezing–thawing processes.

In light of the herein reviewed results, it can be noticed that various spectroscopic methods have tremendous potential for the detection of fraud and verification of several authentication issues in fish and other seafood. Our literature review revealed that the detection of species fraud and thawed fish are the most studied topics, while vibrational spectroscopic techniques, particularly NIR spectroscopy, are the most investigated techniques. Our literature review shows that few spectroscopic studies have been conducted with respect to the determination of geographical origins and detection of the modality of production (capture or aquaculture) of fish and other seafood. The low number of studies regarding authenticity issues, such as geographical origin, may be due to the difficulty associated with modeling variability in the spectra due to challenges related to many factors affecting measurements, such as biological variability, water temperature, and salinity [8,176]. Surprisingly, only a few applications of fluorescence spectroscopy have been reported, although the high sensitivity and specificity of this technique compared to the other spectroscopic techniques is well known. Therefore, fluorescence spectroscopic techniques should be investigated more extensively in future works. 

### 4.2. Meat and Meat Products

*Meat Species Adulteration*: Meat and meat products can have a wide range of market values, depending on several factors. Among other factors, the biological origin is one of the most relevant. In fact, some animals are considered of greater value because of their renowned organoleptic characteristics; consequently, they have a higher selling price. One of the most common adulterations in meat products is the addition of the flesh of a different animal of a lower market value.

In recent years, a lot of effort has been put into developing non-destructive approaches for detecting meat adulterations. In this regard, the choice has often been spectroscopy, especially infrared spectroscopy, which limits or completely avoids any loss of sample material [27] (Table 2). Among the different flesh used as an adulterant, pork, which undesirable for several reasons [29], is probably one of the most investigated and reported materials in the literature. For instance, Kuswandi and collaborators [211] very successfully exploited FT-IR spectroscopy (equipped with attenuated total reflection cell) to detect porcine meat in beef jerky. In order to achieve this goal, the authors exploited three different classifiers, namely LDA, SIMCA, and SVM, and the best results were provided by LDA, giving a total classification rate of 100%. Beside FT-IR, NIR spectroscopy has also been widely exploited in this regard. For instance, Kuswandi et al. used NIR coupled with PLS-DA to detect pork adulteration in beef meatballs [212]. This approach provided extremely satisfying results, since the optimal classification model detected all the adulterated samples. In a similar study proposed by Rady and Adedeji [213], pork adulteration in minced beef was evaluated by NIR spectroscopy combined with PLS-DA. This research provided slightly lower but very promising results. 

After pork, another common adulterant in beef meat is poultry. Several studies have used spectroscopy to detect this kind of adulteration. One example is the work from Deniz and collaborators [214], who demonstrated the possibility of using a fast and non-destructive spectroscopic technique to detect chicken or turkey in beef minced meat. In more detail, adulterated samples of different proportions (5%, 10%, 20%, 40%, and 100%) were prepared and analyzed by FT-IR combined with hierarchical cluster analysis (HCA) and PCA. The data obtained by HCA gave less information than those obtained by PCA, while different spectral bands, especially those of lipids, exhibited noticeable differences between the different meat products (beef, chicken, turkey). A similar study was proposed by Alamprese and collaborators in 2016 [215], who also investigated beef adulteration with turkey, however they inspected fresh, thawed, and cooked meat samples using NIR spectroscopy. Eventually, they used PLS-DA to identify the adulterant and were able to distinguish between samples presenting a low level of adulteration (<20%) and highly adulterated ones (≥20%). 

HSI has been widely used and has shown promise in overcoming the challenges related to measurements of heterogeneous food matrices, such as muscle foods (meat, fish). For instance, Kamruzzaman et al. used this technique coupled with PCA to detect pork [216] and chicken [217] adulteration in beef. Similarly, HSI was applied to detect fraud in minced beef [218]. The data were preprocessed by MSC and SNV, and the performance of two classification models (SVM and RF) was compared. The best results were obtained using the optimized RF model developed on selected wavelengths, achieving an accuracy of 96.87%. 

One of the main advantages of HSI is the possibility to generate a distribution map, allowing the visualization of adulteration levels [14,20]. On the other hand, the data generated from HSI are extremely vast, requiring complex data handling. Multispectral imaging (MSI), however, uses a lower number of spectral bands, thus the acquisition time and complexity of MSI are comparably lower than that of HSI. MSI was successfully used recently in order to detect minced beef adulteration with horsemeat [219]. In this study, the performance of three classification models, namely PLS-DA, RF, and SVM, was explored, and the best results were obtained by the SVM model, giving a correct classification rate of more than 95%. 

Beside spectroscopic methods, the traditional ones (e.g., PCR) are still widely used in this field of quality control. For example, Hou et al. used a PCR method to detect different adulterants (duck, chicken, and goose) in pork, beef, and mutton [132]. Similarly, Kim et al. used it to detect undesired donkey meat in mixtures [131]. Several similar studies have been conducted recently for the same purpose [220,221,222]. Very recently, Yin and co-workers proposed a novel and highly sensitive molecular assay (PCR-based) for the fast revelation of pork components at a concentration of 0.01% in adulterated meat [223]. A relatively novel technique, which is widely used to detect adulterated meat, is DNA barcoding. As an example, Xing et al. successfully exploited DNA barcoding and DNA mini-barcoding to detect mislabeling of several products on the Chinese market [125]. In addition to the previously mentioned approaches, ELISA is another common tool used for species identification in food authentication. For example, it has been used to detect pork-adulterated beef by Mandli and collaborators [141], whereas Perestam et al. compared the performance of the ELISA and of PCR for detecting beef and pork—both approaches have advantages and disadvantages for this purpose [149]. 

**Table 2:** Examples of applications of spectroscopic techniques with respect to various authenticity issues in meat and meat products.

Meat and Meat Products	Authenticity Issue	Analytical Technique	Modeling Method	Reference
Bovine meat	Detection of non-meat ingredients	FT-IR (4000–525 cm^−1^)	PLS-DA, data fusion	[52]
Mutton, beef, pork	Species identification	FT-IR (4000–450 cm^−1^)	SVM, PLS-DA	[73]
Porcine, poultry, bovine, ovine	Species identification	FT-IR (4000–550 cm^−1^)	PCA, PLS-DA, and PLS	[224]
Pig	Identification of feeding regime	Portable NIR (900–1700 nm)	LDA, QDA, and non-parametric Bayes	[225]
Beef, lamb, pork	Species identification	FT-NIR (1100–1938 nm)	One-class classifier partial least squares (OC-PLS), SIMCA	[226]
Pig lard	Origin identification	FT-NIR (750–2500 nm)	PLS-DA	[227]
Lamb, beef, pork	Species identification	HSI VIS/NIR (548–1701 nm)	SVM, CNN	[228]
Beef, meat of rat	Species identification	FT-IR (4000–400 cm^−1^)	PCA, PLSR	[229]
Veal sausages, pork	Species identification	Various FT-NIR equipment	PCA, SVM	[230]
Fresh and rotten beef	Meat identification	VIS/NIR HSI (496–1000 nm)	SVM, LS-SVM, PLSR	[231]
Turkey cuts, processed products	Meat identification	VIS/NIR (400–2500 nm)	PCA, LDA	[232]
Lamb, beef	Species identification	NIR (1100–2300 nm)	PCA, PLS-DA	[233]
Duck, beef, pork	Species identification	NIR (12500–5400 cm^−1^)	DA, PLSR	[234]
Beef, pork, beef heart, beef tallow	Species identification	VIS/NIR (350–2500 nm)	SVM, RF, PLSR, DCNN	[235]
Tan mutton	Detection of thawed meat	NIR HSI (900–1700 nm)	PLS-DA	[236]

PCA, Principal Component Analysis; PCR, Principle Component Regression; LDA, Linear Discriminant Analysis; DA, Discriminant Analysis; QDA, Quadratic Discriminant Analysis; RF, Random Forest; SIMCA, Soft Independent Modeling of Class Analogy; PLS-DA, Partial Least Squares Discriminant Analysis; PLSR, Partial Least Squares Regression; LS-SVM, Least Squares Support Vector Machines; VIS/NIR, Visible–Near-Infrared Spectroscopy; HSI, Hyper Spectral Imaging; FT-IR; Fourier-Transform Infrared Spectroscopy; (D)CNN, (Deep) Convolution Neural Networks.

*Distinction Between Fresh and Thawed Meat*: Beside adulteration with undesired meats, scams concerning meat freshness are unfortunately common, and consequently in the literature it is possible to find different studies aiming to detect this kind of fraudulent action. It is not always easy to discern the freshness of meat by sight, and mislabeling can occur accidentally or intentionally to make illicit profits by selling thawed meat as fresh. Regardless of the reason, it is important to possess suitable tools for the authentication of fresh meat. Once again, in recent years, spectroscopy has played a key role in the detection of this kind of fraud. 

One of the meats investigated the most in this context is chicken, mainly because of the few visual differences that differentiate fresh and thawed products. Nevertheless, Grunert and collaborators have suggested that discrimination can be achieved by FT-IR spectroscopy; in fact, in their study they showed the possibility of using this technique coupled with artificial neural networks (ANN) to discern fresh and thawed samples (frozen and stored for time periods from 2 up to 85 days) [237]. The results were extremely satisfying, since twenty samples (of the twenty-one investigated) were correctly classified. A similar study was proposed by Parastar and collaborators, where fresh and thawed chicken samples were analyzed using a portable NIR instrument and then classified by different methods (random subspace discriminant ensemble (RSDE), PLS-DA, ANN, and SVM); the best results were obtained by using RSDE, providing extremely satisfying results with a classification accuracy higher than 95% [18]. 

*Detection of the Geographical Origin and Production Method*: The traceability of meat and meat products is relevant from different standpoints; for this reason, several approaches have been proposed to assess the origins of meat samples [238]. Traditionally, meat and meat products are traced by means of protein- and DNA-based methods [239]. An example is a recently published paper by Muñoz and collaborators, who focused on Iberian pork meat, which is used to prepare a Spanish typical cured meat product [240]. The authors proposed a single nucleotide variant genotyping panel suitable for recognizing purebreds (Duroc and Iberian) or crossbreds. Interesting solutions for the origin assessment of edible meats were also provided by means of stable isotope ratio analysis. For instance, Erasmus and co-workers showed that δ^15^N and δ^13^C can be used to discriminate South-African lamb breeds in diverse regions [241]. These authors related the isotope abundancies to the pedo-climatic conditions of the different areas. A similar study on a diverse animal species was conducted by Monahan et al., who investigated the possibility of using stable isotope ratio analysis to recognize Irish chickens [242]. Further applications can be found in [243].

Despite the tools mentioned above providing noteworthy outcomes, they are time-consuming, destructive, relatively expensive, and require complex sample preparation. During the first decade of this century (2000–2010), a lot of effort has been put into developing fast and non-destructive spectroscopy-based approaches to achieve the same purpose. However, during the last five years, not many novel strategies have been proposed. For example, recently Zhang and co-authors demonstrated that FT-IR spectroscopy integrated with second derivative infrared spectroscopy (SD-IR) and two-dimensional correlation infrared spectroscopy (2DCOS-IR) coupled with computer vision methodologies represent suitable choices for discrimination of different hams produced in three different locations [244]. 

There are few studies on the potential of spectroscopic techniques for the determination of the production method (dietary background) of meat. One example is a study conducted by Huang and co-authors [245], who applied reflectance spectroscopy in two spectral ranges (400–700 nm and 400–2500 nm) coupled with PLS-DA to discriminate carcasses of lambs reared with 3 feeding regimes, involving perirenal fat from pasture-fed, concentrate-fed, and concentrate-finished after pasture feeding diets. The results demonstrated that the 3 feeding regimes could be distinguished with overall correct classification rates of 95.1% and 99% for the 400–700 nm and 400–2500 nm spectral ranges, respectively. 

*Other Common Adulterants or Contaminants in Meat*: A number of foreign ingredients can be introduced (voluntarily or accidentally) in meat and meat products. Some contaminants can be unintentional, while others are conceived to alter the characteristics of the treated food in order to make it more palatable to the consumers. For instance, the addition of food dyes in meat products is allowed by law, but the types of colorants are strictly regulated; consequently, the possible presence of forbidden dyes has to be checked [243]. Other forms of fraud in meat may involve unwanted or forbidden physical pretreatments, as is the case with irradiation. This practice, which is generally used to extend the shelf-life of food products, is allowed for some foods (for instance dry aromatic herbs) but it is banned for meat. As a consequence, different research studies have been conducted with the aim of developing analytical approaches suitable for the detection of this illicit practice, as in the case discussed by Varrà and co-authors, where irradiated and non-irradiated sausages were discriminated by NIR spectroscopy coupled with orthogonal partial least square–discriminant analysis (OPLS-DA) [246]. 

One further illegal practice is fraudulent mislabeling, consisting of substituting a high-value cut meat with a cheaper alternative, as in the case reported by Sanz and his group [247]. In their study, the authors investigated four different types of lamb muscles using HSI and discriminated the four diverse categories using seven classifiers. The most accurate outcome was achieved using linear least mean squares, which led to a total correct classification rate of 96.67%.

Only limited research has been found in the literature about the use of fluorescence spectroscopy for studying authenticity issues in meat and meat products. In one of the scarce studies, FFFS combined with chemometric tools (PLS and PLS-DA) was successfully applied to classify three different beef muscles, namely *the semitendinosus*, *rectus abdominis*, and *infraspinatus* muscles [248]. These results were confirmed recently in a similar study [95]; in this study, FFFS achieved better accuracy in discrimination of beef muscles than synchronous fluorescence spectroscopy. 

### 4.3. Milk and Dairy Products 

Thanks to its enhanced nutritional value provided by the presence of high-quality protein and minerals, milk is an essential food for people of all ages, from infants to elderly people [249]. Adulteration of milk by the addition of undeclared substances is a widely encountered problem in the dairy industry. Whey, melamine, starch, water, chlorine, formalin, and hydrogen peroxide are the most frequently used adulterants for this type of practice. Mixing milk from different species, replacement of milk fat with non-milk fats or oils, labelling a conventional product as an organic farming product, and false declaration of the processing technology and geographical origin are the other primary fraudulent practices. Several physicochemical methods, liquid and gas chromatography, isotope ratio analysis, and DNA-based techniques have been used for these issues, which involve drawbacks such as having a high cost and being labor-intensive. Spectroscopic techniques (Table 3), being rapid, easy to operate, and applicable to on-line and at-line measurements, as well as providing a high amount of data, are alternatives that can be used to overcome the disadvantages of existing methods [250].

*Addition of Non-Declared Substances*: Urea, melamine, dicyandiamide, sodium bicarbonate, ammonium sulfate, and sucrose are the most frequently used adulteration agents for milk and dairy products [251,252]. Infrared spectroscopy, FT-MIR, and MIRS have been widely applied to determine raw milk and milk powder adulteration by using waste whey [253,254]. In a comprehensive study by Coitinho et al. [67], the FT-IR MilkoScan FT1 device was calibrated and validated using a large number of raw milk samples. Then, the sensitivity (80–90%) and specificity (80–100%) of the method were designated for adulteration of raw milk with different adulterants. Several NIR spectroscopic methods have been utilized to detect milk and milk powder adulteration [255]. In a recent study, a non-targeted method employing benchtop FT-NIR and portable NIR devices coupled with SIMCA was developed to determine eleven potential adulterants in milk powder. The portable device provided lower sensitivity and specificity due to its lower spectral resolution and narrower spectral range [256]. 

**Table 3:** Examples of applications of spectroscopic techniques with respect to various authenticity issues in milk and dairy products.

Milk or Dairy Products	Authenticity Issue	Analytical Technique	Modeling Method	Reference
Yogurt and cheese	Species identification	Front-face fluorescence	PLS-DA and PLSR	[17]
Raw milk	Detection of adulterants	Time Domain NMR	PCA, PLS, and SIMCA	[257]
Milk powder	Detection of adulterants	^1^H NMR	PCA and Conformity Index	[78]
Ultra-heat-treated bovine milk	Detection of adulterants	^1^H and 2D NMR	PLS-DA	[258]
Goat milk	Detection of adulterants	FT-NIR (10000–4000 cm^−1^)	PCA, Q-control, k-NN, SIMCA, and PLS-DA	[255]
Milk powder	Detection of adulterants	NIR (850–2499.5 nm)	PLSR	[259]
Dairy cream	Detection of adulterants	Raman spectroscopy	LDA	[260]
Milk	Species identification	2DCOS-SFS	Relative auto-peak intensity	[261]
Milk	Species identification	NIR (700–2500 nm)	PLS-DA	[262]
Raw and pasteurized milk	Species identification	Raman	PLS-DA	[263]
Milk	Identification of geographical origin	MIR (926–3050 cm^−1^)	GA-LDA	[264]
Cow and goat milk	Detection of adulterants	MIR and Raman	PLSR	[265]
Milk	Species identification	FT-IR (1700–600 cm^−1^)	PCA and HCA	[266]

PCA, Principal Component Analysis; LDA; Linear Discriminant Analysis; DA, Discriminant Analysis; SIMCA, Soft Independent Modeling of Class Analogy; PLS-DA, Partial Least Squares Discriminant Analysis; PLSR, Partial Least Squares Regression; ^1^H NMR, High-Field Nuclear Magnetic Resonance; 2D-NMR, Two-Dimensional Nuclear Magnetic Resonance; FT-IR, Fourier-Transform Infrared Spectroscopy; HCA, Hierarchical Cluster Analysis; (D)CNN, (Deep) Convolution Neural Networks; k-NN, k-Nearest Neighbors; Q-control, Control Chart Q; GA-LDA, Genetic Algorithm Linear Discriminant Analysis; 2DCOS-SFS, Synchronous Fluorescence Spectroscopy coupled with Two-Dimensional Correlation Spectroscopy.

Raman spectroscopy is another vibrational spectroscopic technique that has been widely investigated for adulteration purposes. For example, a portable Raman spectrometer was employed to detect melamine, dicyandiamide, urea, ammonium sulfate, and sucrose adulteration of milk. The standard error of prediction and relative standard deviation values were 39 to 72 ppm and 8% for nitrogen-rich compounds, and 1400 ppm and 10% for sucrose, respectively. The selectivity and efficiency values were 100% for the PLS-DA model in discriminating pure milk samples from adulterated ones [267]. The obtained results were found to be comparable with those of a previous study of the same group, in which a Raman microprobe system was employed [268]. Considering the high-throughput Raman chemical-imaging-based method, it was possible to visualize the spatial distributions of melamine and urea in milk powder and quantify these at the 50 ppm level [82]. Moreover, vegetable oils that were fraudulently added to dairy cream and yogurt were detected by Raman spectroscopy [70,260]. Finding alternative sample preparation procedures is an essential point to be highlighted for efficient Raman spectroscopic analysis in milk and dairy products. Nedeljković et al. [269] performed a preheating process to butter and margarine samples before Raman measurements. In a recent study, the successful use of a portable Raman spectrometer to assess lard adulteration in butter was reported. Samples were melted and mixed thoroughly prior to the Raman measurements [69]. Lohumi et al. developed a line scan spatially offset Raman spectroscopy (SORS) technique that can collect data from packaged butter and margarine samples [270]. 

*Detection of Species Fraud*: Successful discrimination and quantification of milk from undeclared species have been carried out using infrared spectroscopy [271]. Equivalent promising results were reported with Raman spectroscopy [272]. Nonetheless, it is important to emphasize the fluorescence interference problem during Raman spectroscopy measurements, especially with 532 nm lasers. Studies employing lasers with different wavelengths (e.g., 785 and 1064 nm) have extended the use of this technique for milk and dairy product analyses. 

There have been very few studies in the literature reporting the use of NMR for the determination of adulteration. Nonetheless, one study succeeded in discriminating soymilk, bovine milk, goat milk, and their adulterants after coupling chemometrics and metabolite analysis using 1D- and 2D-NMR, with limit of quantification values ranging between 2% and 5% [273]. Some other studies highlighted the changing sensitivity and specificity of the ^1^H time-domain NMR (TD-NMR) method, depending on the used adulterant [81,257]. 

The identification of milk species by employing different measurement techniques involving fluorescence spectroscopy has been studied by several authors [16,274]. Boukria et al. [261] highlighted that cow milk adulteration in camel milk could be detected through the application of the two-dimensional correlation spectroscopy method on SFS spectra. Inclusion of a higher number of samples in the calibration model and scanning of a more comprehensive wavelength range were emphasized as determinant factors in obtaining satisfying discrimination results. 

The successful use of several DNA-based analytical methods has been reported for milk authentication and traceability in the dairy sector [275]. In recent applications, entirely satisfactory limit of detection values were achieved [276,277]. Efforts have been made to develop low-cost and user-friendly PCR devices with accuracy and stability comparable to commercial alternatives [278]. Commercial PCR-based assays designed for the detection and quantitative authentication of animal species in a specific dairy product are also available in the market [279,280].

*Identification of Geographical Origin and Production Method*: Over the last five years, various studies have been reported regarding the authentication of Mozzarella di Bufala Campana Protected Designation of Origin (MBC-PDO) cheese. For example, to combat fraud, Bontempo et al. [281] have successfully proposed the use of the stable isotope method combined with elemental analysis to differentiate both milk and cheese products produced in the PDO area from other products produced outside the PDO area. In another study, Salzano et al. [282] demonstrated that it was possible to distinguish MBC-PDO milk and cheese from non-MBC-PDO products using an advanced GC-MS method and metabolite identification.

Concerning spectroscopic techniques, most of the reported studies were performed in the infrared wavelength range. In more detail, Caredda et al. [264] showed that MIR correctly identified 99% of the ewe’s milk from different geographical regions. In another study, Liu et al. [283] conducted a study to assess the interest in a portable micro-NIR spectrometer to discriminate organic milk from pasture and conventional milk. It was shown that the micro-NIRS device could distinguish between organic and conventional milk as efficiently as the FT-NIRS device (i.e., laboratory device). 

The abovementioned studies prove how frequently spectroscopic techniques are used to detect adulteration of milk and dairy products. Nonetheless, there is an imbalance in use between the different available spectroscopic techniques. Vibrational spectroscopy has been clearly the most preferable applied method used to detect and identify the most common adulterants in milk. However, more studies comparing the performance of NIR, MIR, and Raman spectroscopy for detecting adulteration of milk samples are necessary. Based on the existing literature, it can be noticed that Raman spectroscopy has particular potential for use for routine analysis of milk and dairy products. However, there is still a need for further studies investigating the simultaneous use of adulterants and extending the scope by developing novel untargeted approaches. Regarding the identification or authentication of milk and dairy products based on their geographical origin and processing treatments, surprisingly only a few studies were conducted during the last five years using spectroscopic techniques. This conclusion is similar to that discussed above for fish and meat products. Thus, the use of spectroscopic techniques for differentiation of fresh and frozen–thawed milk and dairy products and investigation of the effects of the applied processes (milk preparation, cheese processing, etc.) or storage conditions that are important for compliance with specifications (such as PDO, protected geographical indication, etc.) are some of the issues that need to be further studied.

### 4.4. Honey and Other Products of Animal Origin 

Honey is a natural sweet product made by bees from the nectar of plants or plant excretions combined with bees’ own specific substances and maturated in the honeycomb. The characteristic flavor, nutritional value, and health benefits of honey depend on its origin and production methods. As a high-quality food product with a high price, honey is often subjected to fraudulent practices, which include mislabeling and adulteration. Development of methods for assessing honey authenticity is of interest to consumers, the honey industry, and food law agencies. Several papers have reviewed the methods used for honey analysis [30,284,285,286,287,288].

*Botanical Origin*: The price of honey strictly depends on its botanical origin. According to botanical origin, honey is classified as unifloral, multifloral (polyfloral), and honeydew [30]. The monofloral honeys are often more expensive than multifloral honeys and are subject to mislabeling or adulteration with cheaper honeys [289].

The most used conventional method for determining honey quality related to its origin is melissopalynological analysis based on the identification and quantification of pollen grains in honey sediment [30]. The physicochemical (profiles) parameters, such as sugars, moisture, proline, and hydroxymethylfurfural (HMF) contents; acidity; electrical conductivity; diastase; and invertase activity are used to establish the origin of a honey. Analytical techniques including gas and liquid chromatography are often used to measure markers of honey origin, such as sugar, phenolic compounds, and flavor compounds. The profiling techniques, stable isotope ratio, and trace element analysis can provide an indication of the geographical origin of honey. The identification of plant species and varieties of honey by DNA fingerprinting is also utilized to assess honey origin.

Spectroscopic techniques have shown considerable potential as rapid and often non-destructive methods used to study the authenticity of honey. In recent years, several studies have demonstrated the potential use of various spectroscopic techniques for evaluation of the botanical origin of honeys (Table 4). For example, NIR spectroscopy and chemometrics were applied to palynological and mineral characteristics of honey collected from Northwestern Spain [290]. Prediction models using a modified PLSR for the main pollen types (Castanea, Eucalyptus, Rubus, and Erica) in honeys and their mineral compositions were established. The ratio of performance to deviation exhibited a good prediction capacity for Rubus pollen and for Castanea pollen, whereas these ratios were excellent for minerals, Eucalyptus pollen, and Erica pollen.

The benefit of data fusion obtained using different analytical techniques was demonstrated for classification tasks of honey according to the botanical origin. The honey samples from three different botanical origins were analyzed by attenuated total reflection IR spectroscopy (ATR/FT-IR) and headspace gas chromatography–ion mobility spectrometry (HS-GC-IMS) [291]. The obtained datasets were combined in a low-level data fusion approach with subsequent multivariate classification by principal component analysis–linear discriminant analysis (PCA-LDA) or PLS-DA. The results showed that data fusion is an effective strategy for improving the classification performance. 

Raman spectroscopy techniques complement information obtained from infrared spectral data and can be used in honey authenticity assessment [287]. Raman spectroscopy, performed using fiber optics, was successfully used to distinguish the botanical origin of unifloral (chestnut, citrus, and acacia) honeys produced in the Italian region of Calabria [292]. Moreover, predictive models were built to quantify important marker indicators in nutraceuticals, such as the main sugars, potassium, and selected sensory properties. 

A promising quick, automatic, and non-invasive approach for honey botanical origin classification was developed using a combination of VIS/NIR hyperspectral imaging and machine learning, namely SVM and k-NN [24]. The developed techniques include noisy band elimination, spectral normalization, and hierarchical classification. The proposed model showed promising results under several classification scenarios, achieving high classification performances. 

The blending of expensive (pure and rare) honey with a cheaper (pure and plentiful) one is another form of honey adulteration. NMR spectroscopy allows the rapid detection of adulterants in honey, as well as the simultaneous quantification of various chemical compounds from a spectrum [287]. For example, ^1^H NMR spectroscopy combined with chemometric techniques was applied to detect and quantify adulteration of acacia honey with cheaper rape honey [293]. The highest prediction accuracy for rape honey addition of −89.7% was obtained using canonical discriminant analysis (CDA), determined from compounds located in the spectral range corresponding to the aliphatic compounds and carbohydrates (3.00–6.00 ppm). Orthogonal projection to latent structure discriminant analysis (OPLS-DA) was used to further discriminate samples of pure acacia honey adulterated with different amounts of rape honey. A PLSR model established a linear fit between the actual and predicted adulterant concentrations, with an R^2^ value of up to 0.9996. 

The fluorescence of honey originates from several groups of compounds, such as amino acids, proteins, phenolic acids, vitamins, fluorescent Maillard reaction products, and other bioactive molecules [23,102,294]. Few studies have demonstrated the potential of fluorescence for authenticity assessment. Fluorescence spectroscopy in EEM mode coupled with parallel factor analysis (PARAFAC) and PLS-DA was applied for classification of honey samples of different botanical origin, including acacia, sunflower, linden, meadow, and fake honey [100]. The classes of honey of different botanical origin were differentiated mainly by emissions from phenolic compounds and Maillard reaction products. PLS-DA constructed from the PARAFAC model provided detection of fake honey samples with 100% sensitivity and specificity. Moreover, PLS-DA classification results gave errors of only 0.5% for linden, 10% for acacia, and about 20% for both sunflower and meadow mixes. 

**Table 4:** Examples of applications of spectroscopic techniques with respect to various authenticity issues of honey.

Honey	Authenticity Issue	Analytical Technique	Modeling Method	Reference
Acacias, lindens, sunflowers, and meadow mixes	Identification of fake honey produced by feeding of bee colonies with a sucrose solution	Fluorescence	LDA	[99]
Honey of various botanical origins, collected from different parts of Ethiopia	Identification of botanical origin	Fluorescence	SIMCA	[101]
Commercial honey from two different provinces of Ecuador	Adulteration	Raman	SIMCA	[295]
Acacia honey	Adulteration of acacia honey with cheaper rape honey	^1^H NMR	CDA, OPLS-DA	[294]
Honey samples (Vitex, Jujube, and Acacia)	Identification of botanical origin	Electronic nose, electronic tongue, NIR, and MIR	PLS-DA, SVM, iPLS	[296]
South African honey	Differntiation between authentic South African and imported or adulterated honey	NIR	PLS-DA	[297]
Honey samples from the Granada Protected Designation of Origin (Spain)	Quantification of the level of adulteration	VIS/NIR	HCA, PCA, LDA, PLS	[298]
High-quality honey (Granada Protected Designation of Origin, Spain)	Identification and quantification of different types of adulterants (inverted sugar, rice syrup, brown cane sugar, and fructose syrup)	VIS/NIR	HCA, PCA, LDA, PLS	[299]
Honey samples belonging to seven different varieties	Identification of botanical origin	FT-NIR HPLC-DAD	PLS-DA	[300]

PCA, Principal Component Analysis; LDA, Linear Discriminant Analysis; SIMCA, Soft Independent Modeling of Class Analogy; PLS-DA, Partial Least Squares Discriminant Analysis; PLSR, Partial Least Squares Regression; SVM, Support Vector Machines; VIS/NIR, Visible–Near-Infrared Spectroscopy; NMR, Nuclear Magnetic Resonance; FT-IR, Fourier-Transform Infrared Spectroscopy; HCA, Hierarchical Cluster Analysis; CDA, Canonical Discriminant Analysis; OPLS-DA, Orthogonal Projection to Latent Structure Discriminant Analysis; iPLS, Interval Partial Least Squares; HPLC-DAD, High-Performance Liquid Chromatography with Diode Array Detection.

*Adulteration Detection*: Honey is a natural product for which the addition of any other substance is prohibited by international regulations. However, due to its high economic value, it is often subject to adulteration. The most common adulterants in honey are sugars from high-fructose corn syrup, corn sugar syrup, inverted sugar syrup, and cane sugar syrup [287]. Adulteration of honey is not limited to direct addition of sugars into natural honey. A common fraudulent practice is overfeeding of bees with concentrated sugar solutions during the main nectar flowing season [30]. Among analytical methods, spectroscopic techniques have become popular for detecting the adulterants in honey [287]. 

FT-IR and PLSR were utilized for the determination of sucrose syrup adulteration of Turkish honeys [301]. The results indicated that the predicted sucrose concentration of honey samples by the spectroscopic method ranged between 4.52 and 15.16%, and that the obtained results were confirmed by chromatography. Several studies reported successful applications of NIR or VIS/NIR spectroscopy for evaluation of honey adulteration. For example, NIR spectra (1300–1800 nm) recorded with a fiber optic immersion probe were used for the detection of high-fructose corn syrup in four artisanal Robinia honeys [302]. The PLSR models developed using the spectral region containing absorption bands related to both water and carbohydrates allowed accurate (root mean squared error of cross-validation; RMSECV = 1.48; R^2^_CV_ = 0.987) detection of the adulterant concentration. Recently, NIR and MIR spectroscopy coupled with SVM and data fusion were utilized to detect adulteration of 20 common honey types from 10 provinces in China [303]. Both pure honey and adulterated samples with different percentages of syrup were analyzed. Compared to low-level data fusion, intermediate-level data fusion significantly improved the detection model, achieving 100% accuracy, sensitivity, and specificity.

Fluorescence excitation–emission spectroscopy was effectively used for the non-destructive and fast detection of fake honey samples obtained during winter feeding of bee colonies with a sucrose solution [99]. Natural honey samples (acacias, lindens, sunflowers, and meadow mixes) were perfectly discriminated from fake honey samples using the developed LDA model. Natural and adulterated honey samples differed significantly in five spectral regions corresponding to aromatic amino acids, phenolic compounds, furosine, and Maillard reaction products.

Eggs are consumed worldwide and are well known as a source of vitamins, minerals, phospholipids, and high-quality proteins. EU regulation classifies egg production into four hen housing systems, including 0 for organic production, 1 for free range, 2 for barns, and 3 for cages. Consumers are willing to pay higher prices for eggs produced in a way that considers animal welfare [304], and chicken eggs are often a subject of food fraud. Therefore, there is a need for analytical methods that are suitable for classifying eggs and for detecting the fraudulent mislabeling of eggs obtained from different production systems. 

Various procedures are used to discriminate eggs, including carotenoid profiling, fatty acid composition, and mineral content procedures. Eggs from various systems (1-, 2-, and 3-coded eggs) may be discriminated through fluorescent patterns on egg surfaces or stable nitrogen isotope compositions. Stable isotopes methods were used to develop authentication criteria of eggs laid under cage, barn, free range, and organic farming regimens [305]. Recently, discrimination of selected chicken eggs in China’s retail market based on multielement and lipidomic analyses was reported [306].

UV-VIS/NIR spectroscopy and chemometrics were utilized for a complete detection of the housing systems declared on the eggs’ label [307]. Eggs were perfectly classified into the four housing systems by applying quadratic discriminant analysis for UV-VIS/NIR spectra of the yolk lipid extracts. NMR spectroscopy was successfully utilized as a tool to screen eggs according to the different systems of husbandry [304]. In this study, ^1^H NMR of freeze-dried egg yolk samples were analyzed using PCA followed by a linear discriminant analysis (PCA-LDA). The prediction model allowed for the correct classification of about 93% of the organic eggs, barn eggs, and free range eggs.

## 5. Challenges and Future Trends

Even though extensive research regarding the authenticity and detection of fraud by on-site and real-time approaches has been carried out in recent years, several key challenges still remain concerning both technique-related issues and the model validation framework.

Regardless of the non-destructive approach considered, the correct sampling procedure is pivotal to provide valuable information, and thus to embrace the complexity of modern food authentication [308]. Indeed, non-destructive approaches include non-targeted methods (i.e., fingerprint techniques) with the ability to detect multiple small modifications in the considered food product and to extract these modifications as relevant information using the proper multivariate statistic approach. However, the database used to address the authentication issue should consider the main sampling-related criteria, such as the definition of the sample unit, number of samples, sample variability, handling procedure, representativeness, and so on. The most important considerations that must be addressed when creating a food authenticity database are discussed in the position paper by Donarski and co-authors [309]. These issues are highly relevant, as the database is used to define an “authentication rule”, which is applied to compare the unknown sample fingerprint with those of authentic reference samples [308]. Even though the creation of the foodstuff-specific database was done considering the perfect sampling procedure and can quickly cover the variability expected from test samples, continuous maintenance of the database is needed to ensure long-term ability to return reproducible results, and most of the scientific publications do not meet this requirement.

Once the authentication issue has been defined and the database creation has been designed accordingly, consideration needs to be given to the definition of a standard operating procedure (SOP) from the sample preparation to the analytical protocol. 

DNA-based methods, protein-based methods, and isotopic techniques require specific consideration when defining SOP. Indeed, in these cases, the required analytical steps for sample preparation highly influence the results and their interpretation [2]. As for any analytical technique, different experimental factors can influence the obtained results, introducing an analytical deviation that is not related to the authentication issue under study. These deviations should be reduced to the lowest terms and controlled to ensure that they do not introduce confounding results in the analysis [309]. The influence of experimental factors cannot be avoided, even in spectroscopic technologies (e.g., vibrational spectroscopy, NMR, and fluorescence spectroscopy), despite being reproducible and barely influenced by changes in sensitivity over time. Indeed, they do not generally require any sample preparation, guaranteeing long-term stability and online or in-line application along the production chain. This is particularly true for liquid “homogeneous” samples, whereas solid heterogeneous products, such as meat, fish, and dairy products, may require moderate sample preparation or multiple point measurements. Moreover, the choice of the proper acquisition mode is fundamental to obtain reliable spectroscopic results according to the nature of the food product, including the type of radiation (NIR, IR, NMR, or fluorescence spectroscopy), sample presentation (transmission, absorbance, reflectance, excitation or emission fluorescence, synchronous fluorescence, EEM), type of sample holder (cuvette, fiber probe, attenuated reflectance holder, integration sphere), and working temperature, among others. 

Actually, HSI technologies are a valid alternative to point spectral scanning, whereby the spatial distribution of components in heterogeneous products can be distinguished using site-to-site spectroscopic fingerprint specificity. Food quality and authenticity, especially referring to meat products, have been widely investigated by HSI technologies associated with NIR radiation. However, most of the reported works are feasibility studies at the laboratory scale, whereas there is a lack of studies proving the model’s robustness at the processing plant level. Furthermore, the huge disadvantages of HSI technology are related to the large amount of produced data for each single measure and the relatively long processing times for these data. However, simplified instruments (multispectral imaging systems) developed for specific applications could reduce the spectral range to be scanned to a few selected wavelengths, thus minimizing both the acquisition time and generated data, which could be managed quickly with the proper ad hoc chemometric method [310]. Simplified, miniaturized, and portable instruments have been developed for the whole spectroscopic field, which are oriented toward food authentication [311]. Certainly, the performance of these instruments in terms of the electromagnetic range covered, resolution, signal-to-noise ratio, specificity, and sensitivity is lower if compared to the results obtained by benchtop instruments [198]. However, their use for ad hoc authentication purposes and their combination with robust chemometric algorithms for classification applications are expected to be major trends in the coming years. 

As described in Section 2, multivariate data analysis is the fundamental step taken to produce a model able to classify samples as authentic or non-authentic from any emerging detection method result. No matter the algorithm used to solve an authentication issue, robust validation of the model is mandatory to guarantee reliable and reproducible results and to favor the acceptance of these methodologies in legislation. This theme is quite contentious, and it is one of the major reasons for the refusal of emerging detection methods, along with the standardization procedures [2,60]. Although several attempts have been made to meet the need for common and reliable validation protocols, there is still a lack of validation programs for method developers, which is also reflected in the scientific literature. In the paper by Oliveri [46], a detailed analysis of the key aspects of model evaluation is discussed. This paper could be a landmark when defining a global workflow to solve an authentication issue using spectroscopic techniques.

Thus, it is undeniable that spectroscopic techniques have enormous advantages over the targeted approaches when addressing a food authentication issue; however, their wide application outside of laboratories remains challenging. Meeting these challenges will align emerging spectroscopic methods with the needs of food fraud risk management systems, paving the way for their use for food integrity assurance, such as with the EU-wide Rapid Alert System for Food and Feed (RASFF).

## 6. Concluding Remarks

This paper has reviewed and discussed papers published in the last 5 years on the use of different analytical methods used to target issues related to fraud in both food and products of animal origin. The available literature in the field has shown an increase in the number of applications combining rapid analytical methods (e.g., DNA analysis, vibrational spectroscopy) with modern data analytics (e.g., multivariate data analysis). The body of research as a whole presents indisputable evidence that these methods and techniques have enormous advantages over other approaches when addressing food authentication. However, several challenges still exist related to the wide application and implementation of these technologies in both research and commercial laboratories. This calls for the need for a continuous exchange between the food authentication stakeholders, together with the growth of a new generation of scientists able to work in both academic and industrial environments and who are skilled in facing all aspects of food authentication using non-targeted techniques.
